# LASP1 in Cellular Signaling and Gene Expression: More than Just a Cytoskeletal Regulator

**DOI:** 10.3390/cells11233817

**Published:** 2022-11-29

**Authors:** Elke Butt, Cory M. Howard, Dayanidhi Raman

**Affiliations:** 1 Institute of Experimental Biochemistry II, University Clinic Wuerzburg, 97080 Wuerzburg, Germany; 2 Department of Cell and Cancer Biology, College of Medicine and Life Sciences, University of Toledo, MS 1010, Toledo, OH 43614, USA

**Keywords:** LASP1, AKT, CXCR4, structure, cytoskeleton, phosphorylation, transcriptional regulation, epigenetics, nucleus

## Abstract

LIM and SH3 protein 1 was originally identified as a structural cytoskeletal protein with scaffolding function. However, recent data suggest additional roles in cell signaling and gene expression, especially in tumor cells. These novel functions are primarily regulated by the site-specific phosphorylation of LASP1. This review will focus on specific phosphorylation-dependent interaction between LASP1 and cellular proteins that orchestrate primary tumor progression and metastasis. More specifically, we will describe the role of LASP1 in chemokine receptor, and PI3K/AKT signaling. We outline the nuclear role for LASP1 in terms of epigenetics and transcriptional regulation and modulation of oncogenic mRNA translation. Finally, newly identified roles for the cytoskeletal function of LASP1 next to its known canonical F-actin binding properties are included.

## 1. Introduction

### 1.1. Structural Features of LASP1

**Figure 1:**
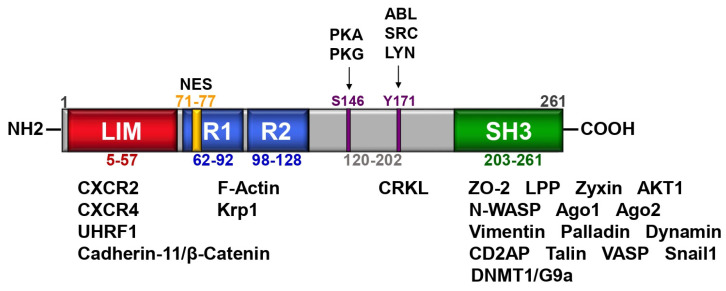
Graphic illustration of LASP1 domain structure. LASP1 consists of a LIM (LIN-11, Isl-1, and MEC-3) domain (red), two F-actin binding nebulin repeats (R1 and R2) (blue), a linker site with two key regulatory phosphorylation sites (S146 and Y171) (purple), and a SH3 (Src Homology 3) domain (green). The first nebulin repeat includes a nuclear export signal (NES) between residues 71–77 (yellow). Known domain-specific protein–protein interactions are provided.

The LIM and SH3 protein 1 (LASP1) was first identified in lymph nodes of breast cancer patients in 1995 [1]. The gene is located on chromosome 17q11-21.3 and encodes a protein of 261 amino acids (aa). The protein expression level of LASP1 was found to be upregulated in several tumor types (reviewed in [2]), except melanoma [3]. Structural analysis of LASP1 revealed a protein composed of a N-terminal LIM-domain with two zinc finger motifs, followed by two central actin-binding nebulin repeats [4,5], flanked by a linker region and a C-terminal SH3 domain, known to bind to several proline-rich segment proteins (Figure 1). The first nebulin repeat includes a nuclear export signal (NES; aa 71–77) [6]. A recent molecular evolutionary analysis of vertebrate and invertebrate lasp family proteins revealed that all proteins harbor the same ancestral gene and that the LIM domain, nebulin repeats and SH3 domain are highly conserved between both groups, although the invertebrate lasp linker sequences vary [7]. 

**Figure 2:**
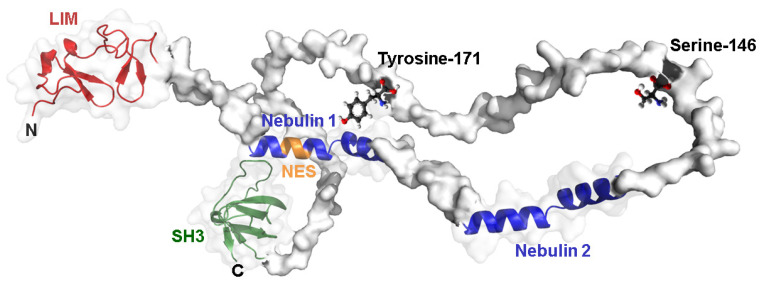
LASP1 3D structure. Three-dimensional structure prediction of LASP1 highlighting sites at serine 146 and tyrosine 171. The structure was designed using Alphafold https://alphafold.ebi.ac.uk/entry/Q14847; accessed on 4 October 2022.

A 3D structure prediction of LASP1 illustrates two key phosphorylation sites in the linker region that face the two nebulin repeats which may have a regulatory function (Figure 2). First, phosphorylation of LASP1 at serine 146 (S146) is regulated by cAMP- and cGMP-dependent protein kinases (PKA and PKG) [8]. Tyrosine 171 (Y171), located opposite of the nebulin repeat, is phosphorylated by various cytosolic tyrosine kinases such as c-*Src* [9], ABL [10] and LYN [11] (Figure 1 and Figure 2). Interestingly, phosphorylation at one site hinders phosphorylation at the other site [11].

**Figure 3:**
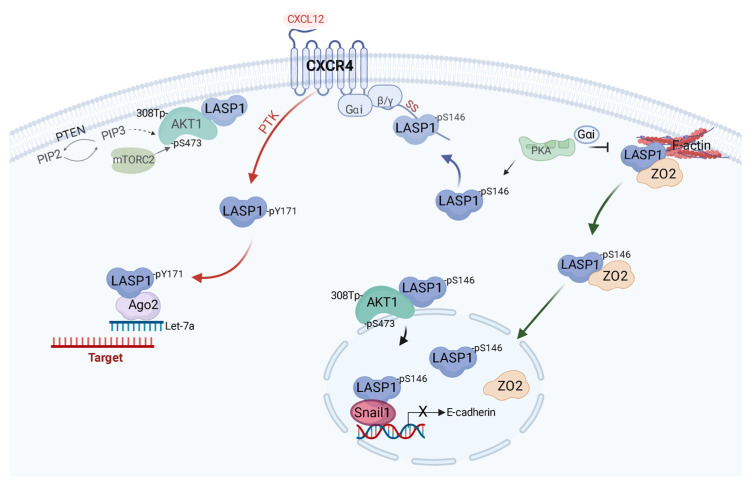
Phosphorylation-dependent regulation of LASP1 in TNBC. Function of LASP1 in TNBC cells is primarily regulated by its phosphorylation status at two specific sites, serine 146 (pS146) and tyrosine 171 (pY171). Unphosphorylated LASP1 primarily binds to F-actin and is complexed with other proteins such as zona occludens protein 2 (ZO2). Activation of protein kinase A (PKA) by either growth receptor signaling or Gα_s_ pathways lead to phosphorylation of LASP1 at S146. pS146-LASP1 then preferentially binds to the C-terminal tail of chemokine receptor CXCR4 (implicated in breast cancer metastasis). Concurrently, pS146-LASP1 anchors AKT1 to the cell membrane serving as a scaffold and a facilitator of signaling between PTEN and mTORC2. Some pS146-LASP1 also dissociates from F-actin and is transported to the nucleus in a ZO2-dependent mechanism. Within the nucleus, pS146-LASP1 associates with proteins such as Snail1 which increases the stability of this transcriptional factor and promotes epithelial-to-mesenchymal transition through repression of epithelial markers such as E-cadherin. Once CXCR4 is activated, a fraction of LASP1 is preferentially phosphorylated at Y171 by phosphotyrosine kinases (PTK), inhibiting its interaction with the CXCR4 C-terminal tail. Concomitantly, G-protein α_i_ is released, inhibiting PKA and potential for S146 function. pY171-LASP1 facilitates other aspects of the metastatic cascade through interactions with other proteins such as the RNA-induced-silencing-complex via binding to argonaute protein (Ago2). Pathway was created using BioRender software: https://biorender.com; accessed on 12 October 2022.

The functions of LASP1 are based on two pillars; (1) scaffolding binding and (2) phosphorylation. Protein phosphorylation is one of the most common post-translational modifications and may convert the site around phosphorylation from hydrophobic (apolar) to hydrophilic (polar) which triggers conformational changes leading to either attraction or releases of proteins simply due to the introduction of the negative charge by the phosphate group [12]. Throughout this review, we provide insight on the regulatory function of the phosphorylation status of the LASP1 and specific domain interactions (if known). A summary of LASP1 and its phosphorylation status on cell signaling events in triple-negative breast cancer (TNBC; negative for estrogen, progesterone and HER2 receptor) is provided (Figure 3).

### 1.2. LASP1 Expression

LASP1 is detected in all non-muscle tissues with highest protein levels in the gastrointestinal tract (https://www.proteinatlas.org/ENSG00000002834-LASP1; accessed on 30 September 2022) and is localized in actin-rich subcellular regions such as focal adhesions and lamellipodia. Expression is regulated by several nuclear factors. Preliminary database analysis identified a p53 response element in the LASP1 gene promoter and this was later confirmed in hepatocellular carcinoma (HCC) where LASP1 is upregulated by p53 repression [13]. However, this finding does not translate for all cancer types as p53 is often deleted or mutated in cancer [14]. There is evidence for LASP1 upregulation in tumors under hypoxia [15] as a hypoxia response element has been identified in the LASP1 promoter region and was shown to stimulate LASP1 expression in pancreatic cancer cells in vitro and in mouse tumor xenografts [16]. On the contrary, in trophoblasts, the hypoxia effect is indirect by inducing miRNA-218 to downregulate LASP1 protein levels [17].

In recent years, there is a plethora of misinformation concerning the regulation of LASP1 by miRNAs with several papers being retracted. We therefore avoid the discussion of LASP1regulation by miRNAs in this review [18]. An earlier review by the authors summarizes some of the miRNAs that lead to upregulation of LASP1 in specific tumor types [2].

## 2. LASP1 and Cellular Signaling

### 2.1. Chemokine Receptor Signaling

Chemokine signaling is essential for coordinated cell migration in health and disease. Typically, chemokines signal through heptahelical, G protein-coupled receptors [19]. LASP1 interacts with the LKIL motif (aa 327–330) at the intracellular C-terminus of the chemokine receptors CXCR2 and CXCR4 which are overexpressed in breast cancer [20]. Direct binding to CXCR4 requires LASP1 phosphorylation at S146 by PKA. This presumably stabilizes CXCR4 receptor and blocks degradation by sterically hindering phosphorylation at multiple PKC or GRK serine phosphorylation sites around the LKIL motif that are important for receptor internalization and deactivation [21]. Chemokine receptor stimulation by CXCL12 releases the PKA-inhibitory heterotrimeric Gαi-protein complex and activates c-*Src* family of tyrosine kinases. As an effect, LASP1 becomes phosphorylated at Y171 while S146 phosphorylation decreases (gets dephosphorylated) that is concomitant with lack of association of LASP1 with chemokine receptor CXCR4 [11].

Breast cancer cells show high levels of LASP1 phosphorylated at S146 by PKA, while in chronic myeloid leukemia (CML) cells a dominant phosphorylation of LASP1 at Y171 by the constitutively active BCR-ABL tyrosine kinase was observed [11]. The CXCR4 receptor is downregulated, and no binding or stabilization is expected. When BCR-ABL tyrosine kinase is inhibited, the levels of pY171-LASP1 plummeted, concomitant with an increase in pS146-LASP1 levels, and an upregulation and stabilization of the CXCR4 receptor in these cells [11]. pY171-LASP1 also binds to Crk-like protein (CRKL) [22] in CML cells and may regulate BCR-ABL signaling.

### 2.2. PI3K/AKT/mTOR

The PI3K/AKT/mTOR pathway is an intracellular signaling pathway that plays a pivotal role in the cell cycle control and survival. It is altered in several primary tumors, with AKT being overexpressed and hyperactivated by phosphorylation. PI3K binds to the PH domain of AKT and induces conformational changes and the subsequent phosphorylation of AKT. Activated AKT moves from the cytoplasm to the cell membrane and directly or indirectly activates its downstream molecular proteins such as mTOR [23].

**Figure 4:**
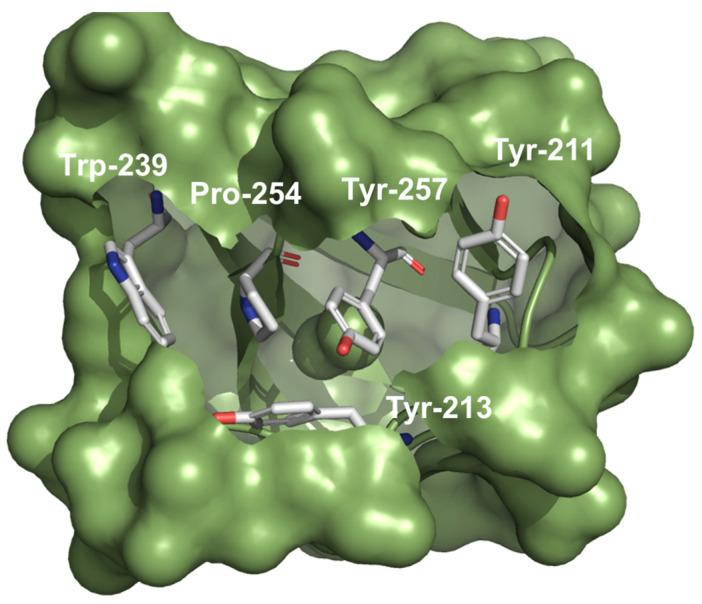
LASP1-SH3 domain. Coulombic surface presentation of the LASP1-SH3 domain illustrating a negatively charged cleft and subsequent proline motif binding by conserved aromatic amino acids. The structure (PDB3i35) was designed using PyMol Molecular Graphic System Version 2.5.4 open source software (Schrödinger, LLC, New York, NY, USA): https://pymol.org; accessed on 12 October 2022.

Lately, a direct (or indirect) interaction between the catalytic, proline-rich C-terminal binding region of AKT1 and the LASP1-SH3 domain was observed, with a higher affinity of AKT1 for pS146-LASP1 and an impaired binding to pY171-LASP1 in comparison to the unphosphorylated protein [11]. The LASP1-SH3 domain modulates a negatively charged cleft (Figure 4) that interacts with the N-terminal proline-rich sequence, flanked by positively charged residues in AKT1 [11]. So far, 11 proline-rich proteins have been identified to bind to the LASP1-SH3 domain (Figure 1, and reviewed in [2,24]). Immunofluorescent analysis showed LASP1-AKT1 co-localization at the cell membrane while pS146-LASP1/AKT1 co-localized at the perinuclear area–particularly in endosomes (probably Rab11a-positive pool) [11]. The authors hypothesize a scaffolding model with AKT1 bound to LASP1 at the cell membrane, in close contact to PIP3 and mTORC2 complex facilitating AKT1 phosphorylation. In addition, LASP1-dependent regulation of the tumor suppressor PTEN, opposing the activity of PI3K, was reported [25]

Recently, pharmacological network analysis revealed that several ingredients of a traditional Chinese medicine (TCM) against atherosclerosis decreases PI3K/AKT signaling [26]. Molecular docking simulation exhibited strong binding of several of these TCM bioactive ingredients towards the LASP1-SH3 domain, suggesting a blocking of AKT binding to this domain, and hence a reduced AKT1 phosphorylation and activation [26].

The LASP1-AKT interaction was mainly studied in the context of breast cancer [11,27]. Phosphorylation of LASP1 by uncontrolled activation of PKA/cAMP signaling in tumor cells [28] reduces LASP1 affinity to F-actin [8]. Concurrently, pS146-LASP1 can serve as a scaffold in AKT signaling. Phosphorylation of both proteins allows for their translocation into the nucleus. When PTK–mediated signaling pathways are activated in these cells, pY171-LASP1 predominates which reduces LASP1’s affinity for AKT1 and facilitates other functions. Furthermore, overexpression of LASP1 enhanced AKT1-S473 phosphorylation in combination with a decreased E-cadherin expression and epithelial-mesenchymal transition (EMT) while, in return, LASP1-depletion resulted in reduced pS473-AKT1 phosphorylation. This is not only observed for breast cancer but, so far, also in glioma, lung, prostate, and colorectal cancer [29,30,31,32].

Recently, LASP1 interaction with HER2 in ovarian cancer cells was demonstrated, however, no detailed domain binding analysis was performed but the authors claimed a LASP1 phosphorylation-independent association [33]. Our own sequence analysis identified several putative proline-rich domain motifs pointing to a SH3 domain interaction. The functional consequences of the LASP1-HER2 and downstream PI3K/AKT signaling will need further investigation.

### 2.3. β-Catenin

The cadherin–catenin adhesion complex is the central component of the cell–cell adherens junctions (AJ) that transmit mechanical stress from cell to cell [34]. Binding of the Cadherin-11/Catenin complex to the LASP1-LIM motif was visualized and confirmed in AJ of synoviocytes. Patients with rheumatoid arthritis showed increased LASP1 levels while LASP1 deficiency altered the cell-to cell contacts and was associated with a less destructive phenotype [35]. Due to LASP1’s interaction with β-Catenin, LASP1’s role in this signaling pathway will need to be elucidated.

## 3. LASP1 and Gene Regulation

### 3.1. Nuclear Import

Nuclear localization of LASP1 was first described in breast cancer and was correlated with tumor progression, metastasis, and a reduced overall survival of the patients [14]. Nuclear presence and poor outcome was also observed in hepatocellular carcinoma [36], chordoma [37] as well as in prostate [38], colorectal [39], lung [32], and bladder cancers [40]. Despite a nuclear export signal, LASP1 is reliant on a nuclear shuttle partner–the tight junction protein zona occludens 2 (ZO2) and potentially AKT1. Phosphorylation of LASP1 at S146 releases the protein from the cytoskeleton (shown for actin and zyxin) and allows the re-localization of the LASP1-ZO2 complex from the outer cell membrane into the nucleus [7]. In contrast to solid tumors, CML cells show low levels of ZO2 and therefore no nuclear LASP1 localization was detected. LASP1 nuclear import is also regulated by another cytoskeletal protein, Talin. Talin binds to the SH3 domain and substitutes for ZO2 and thereby inhibits nuclear LASP1 transportation as shown in endometrial cells [41].

### 3.2. Matrix Metalloprotease Regulation

Matrix metalloproteases (MMPs) are calcium-dependent zinc-containing endopeptidases that are capable of degrading extracellular matrix proteins (ECM) such as collagen, elastin, and fibronectin. These proteins are necessary for tissue remodeling, cell proliferation, migration (adhesion), local invasion and therefore cancer metastasis [42].

In this respect, LASP1 plays a pivotal role on expression and secretion of MMPs. In human macrophages, LASP1 is localized in the ring structure of podosomes. Knockdown of LASP1 decreased matrix degradation capacity in these cells [43]. In TNBC, silencing of LASP1 reduced gene expression levels of MMP9 and 1 [44]. This was further supported by reports showing a reduced MMP1, 3 and 9 expression in MDA-MB-231 breast cancer cells after stable LASP1 knockdown. Mechanistically, an effect of LASP1 on the prevalent MMP transcription factor AP-1 was suggested and was verified by luciferase reporter assays [45]. An analysis of LASP1 regulated genes in LASP1-depleted breast cancer and hepatocellular carcinoma, revealed a disproportionality high regulation of AP-1 controlled protein expression, and supported the regulation of AP-1 by LASP1 [45,46]. Reduced MMP1 levels after LASP1 depletion were also observed in LNCaP prostate cancer and T24 bladder cancer cell lines [45] suggesting a general role of LASP1 in favoring distant metastasis by enhanced transcription and secretion of MMPs through invadopodia.

### 3.3. Snail1 Stability

Snail1 is a labile transcription factor and plays a pivotal role in EMT. Blockade of the ubiquitination and degradation of Snail1 lowers the gene expression of adherens junction proteins such as E-cadherin; increases N-cadherin and fibronectin expression and these events promote cell invasiveness [36,44,47]. A proteomics study initially revealed a LASP1-Snail1 interaction and was validated with a proximity ligation assay [44]. This interaction was further studied in the context of CXCR4 activation in TNBC [25]. After CXCR4 activation, LASP1 is preferentially phosphorylated at Y171 However, LASP1/Snail1 interaction experiments with phosphomimetic LASP1 pulldown assays revealed that LASP1 phosphorylation status did not notably regulate LASP1-Snail1 association in vitro [25]. Additionally, activation of CXCR4 increased the levels of Snail1 via PI3K-dependent phosphorylation/activation of AKT and concomitant phosphorylation/inactivation of GSK-3β, a protein known to target Snail1 for ubiquitination/degradation. Moreover, A20 and LSD1 levels increased following CXCR4 stimulation, and further assist in Snail1 stability [25]. Based on ChIP analysis, LASP1 binds to the E-cadherin promoter in a CXCL12-dependent manner, indicating a functional role for LASP1 in Snail1 stability by scaffolding Snail1 to the promoter sites of E-cadherin [25].

The question remains whether LASP1 can directly regulate transcription by interactions with DNA. Proving of a direct DNA binding via the two LASP1 zinc finger motifs by ChIP assay failed (unpublished results of the authors). Although known for zinc fingers in general, the two zinc finger motifs in the LASP1-LIM domain are not involved in homodimerization [48] and no direct interaction with DNA has been observed [45].

### 3.4. Epigenetic Modulation

Epigenetics represents another critical avenue of transcriptional dysregulation in cancer cells. An example is aberrant DNA methylation. UHRF1 (E3 ubiquitin-protein ligase) is thought to recruit the DNA methyltransferase DNMT1 to the replication fork [44]. Using proteomics, pulldown, and co-immunoprecipitation approaches, binding of the LASP1-LIM domain to UHRF1, in association with DNMT1 and histone methyltransferase G9a was shown and may regulate chromatin structure and gene expression at late G1 and G2/M phase [44]. This represents another potential transcriptional regulation mechanism by LASP1.

### 3.5. RNA-Induced Silencing Complex

As described above, the activation of chemokine receptors in breast cancer shifts the pS146-LASP1 landscape to one that is predominated by pY171 on LASP1. In TNBC, the change in the phosphorylation status of LASP1 in response to CXCR4 activation then allows LASP1 to interact with another protein: Argonaute-2 (Ago2). Pulldown experiments revealed Ago2 binding to LASP1 through its LIM and SH3 domains (preferentially after LASP1-Y171 phosphorylation) [46]. Non-phosphorylated LASP1 and pS146-LASP1 show weak to no association with endogenous Ago2 [49].

Argonaute proteins are part of the RNA-induced-silencing-complex (RISC), which plays a central role shaping the transcriptome through RNA interference [50]. After binding to mRNA, Ago2 cuts the complementary strand via an endonuclease activity into single-stranded siRNA/miRNA [51]. In this respect, pY171-LASP1 interaction with Ago2 promoted expression of Let-7a miRNA proteins such as CCR7, eIF4G2 and cyclin D1, which are involved in tumor progression, lymph node metastases, therapy resistance and distal metastases to the visceral organs [49]. The exact mechanism by which LASP1 affects Ago2 activity (such as prevention of target binding, hindering binding to mRNA, or blocking enzymatic activity) are currently under investigation. This data suggests that activation of one type of chemokine receptor, i.e., CXCR4 by CXCL12 leads to expression of another chemokine receptor CCR7 through the scaffolding activity of LASP1. Both CXCL12-CXCR4 and CCL21-CCR7 receptor axes are highly involved in the metastatic cascade.

### 3.6. Eukaryotic Initiation Factor 4F Complex

The eukaryotic initiation factor 4F complex is a complex consisting of the scaffold eIF4G, cap binding protein eIF4E, mRNA helicase eIF4A, and modulation protein eIF4B [48]. This complex is involved in the rate limiting step of protein translation which prepares the mRNA for ribosome recruitment. Using purified proteins and a proximity ligation assay, LASP1 was shown to directly interact with both, eIF4A and eIF4B in a CXCL12-dependent manner [52]. When LASP1 was stably knocked down, the downstream targets of eIF4F such as CCND1, BIRC5, and MDM2 were reduced in TNBC cells [52]. This work could suggest that LASP1 can further modulate the gene expression at the level of protein synthesis. Domain or phosphomimetic binding was not determined in the study and these aspects of LASP1 binding will need to be further explored.

## 4. New Insights on the Cytoskeletal Function of LASP1

### 4.1. Cytoskeletal Binding

LASP1 is predominantly involved in the reorganization of cytoskeleton during cell motility and localized at the plasma membrane and in actin-rich subcellular protrusive structures such as lamellopodia, filopodia, pseudopodia, and invadopodia [43,53]. Binding to F-actin occurs via the SH3 domain and the first nebulin repeat [54] while no binding to G-actin was observed [8]. Phosphorylation of LASP1 at S146 in human LASP1 [8] and T156 in mouse Lasp1 [48] by PKA and PKG causes reduced F-actin-binding and a more cytosolic localization of the protein.

### 4.2. Bone Development

In mice, Lasp1 is expressed in the growth plate, specifically in resting and hypertrophic chondrocytes [55]. However, Lasp1-deficient mice had slightly lower body weight but developed normally without defects in skeletal development [56]. Recent research has focused on the protein in osteosarcoma. Biopsies revealed strong LASP1 expression in chordoma and show low expression in less malignant chondrosarcoma [37] supporting the known function of the protein on cell proliferation and migration as seen in other tumor types.

### 4.3. Neuronal Expression

Newfound roles for LASP1 are continually being discovered secondary to its ability to regulate the F-actin cytoskeleton. For example, the LIM domain and the nebulin repeats work cooperatively to target LASP1 to the sites of active actin polymerization in protruding lamellipodia of developing axons [57] and to stabilize the actin filaments in dendritic spines [58]. LASP1 is concentrated in synaptic sites of hippocampal neurons, suggesting a functional role in synaptic transmission [59]. This finding is supported by the proteomic identification of LASP1 in postsynaptic preparations of rat brains [60]. In all, these discoveries support the hypothesis that LASP1 is necessary for both the development and maintenance of neuronal circuitry.

### 4.4. Kidney Function

LASP1 may also be necessary for proper kidney function. Podocytes are specialized epithelial cells that wrap around the glomerular capillaries and are part of the filtration unit of the kidney. In podocytes, LASP1 is crucial for the slit membrane integrity and glomerular filtration. Activation of the renin-angiotensin-aldosterone system by Ang II, significantly increased pS146-LASP1 phosphorylation by PKA and resulted in a re-localization of the protein from along intracellular actin stress fibers to the lamellipodia at the outer cell membrane thereby anchoring slit membrane components like CD2AP to the actin cytoskeleton through an interaction with the SH3 domain [61]. Nephrocyte-specific knockdown of Lasp in Drosophila melanogaster showed reduced number of slit membranes and mislocalization of F-actin [61]. This is in agreement with a second study by Artelt et al., showing that a podocyte-specific knockdown of Palladin, a LASP1 binding partner along actin stress fibers [62], leads to a decreased pLasp1 phosphorylation and morphological deviations like an enlarged sub-podocyte space [63].

## 5. Conclusions

**Figure 5:**
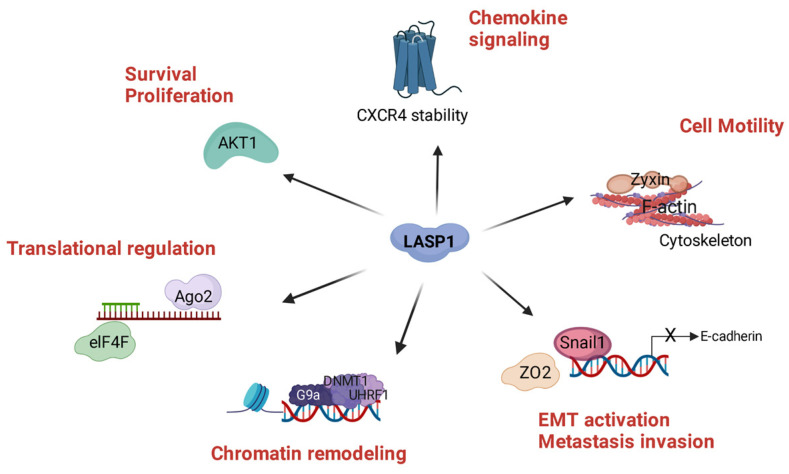
LASP1, a Hub for Cancer Progression. LASP1 is a multifaceted protein in cancer progression and metastasis through several direct and indirect protein interactions. LASP1 binds to F-actin and is localized to actin rich protrusive structures such as invadopodia and lamellipodia, necessary for invasion and metastasis. Cancer cell viability and proliferation are promoted through activation of the PI3K/AKT signaling pathway, presumably through the LASP1 scaffolding function at the cell membrane. LASP1 plays a role in chemokine signaling by modeling CXCR4 stability, a receptor also overexpressed in several cancer entities. Nuclear LASP1 presence promotes metastasis by stabilizing Snail1 and enhancing epithelial-mesenchymal transition (EMT) and by remodeling chromatin to alter the transcriptome. Expression of mRNA is regulated by the interaction of LASP1 with Ago2, a protein of the RNA-induced silencing complex (RISC) and with eukaryotic initiation factor 4F complex (eIF4F) which induces translation of mRNA into oncoproteins. Created with BioRender software: https://biorender.com, accessed on 18 November 2022.

In this review, we summarized the newest data on LASP1 signaling, especially the phosphorylation-dependent binding interactions and gene expression regulation. After the initial identification of LASP1 in the lymph nodes of breast cancer patients in 1995, investigations over the last 20 years suggested a critical role of the protein in cancer biology, mainly in tumor progression and the metastatic cascade including currently defined roles in chemokine receptor regulation, AKT signaling, Snail1 stability, and modulation of gene expression levels (Figure 5). The function is further expanded to normal cellular physiology, especially the LASP1 binding to F-actin, and the currently investigated roles in kidney function, axon signaling, and bone development. This confirms that LASP1 is not only a critical F-actin cytoskeleton regulator, but also a complex, multifaceted signaling adaptor protein.

The precise mechanisms of the latter roles will need to be studied further. The nuclear role of LASP1 is still rudimentary and far beyond just stabilizing DNMT1 and Snail1. There is also evidence for a role in AP-1 transcriptional regulation [45]. In some aspects, LASP1 and “LASP1-regulated proteins” might just share similar shRNAs.

In hematopoietic cells, LASP1 is not localized to the nucleus assuming an additional cytosolic role of the protein in CML tumor cell persistence and proliferation [64]. An eIF4F and RISC-mediated gene regulation, modulated by LASP1 interference with the endonuclease activity of Ago2 and the helicase activity of eIF4A, possibly via phosphorylation-specific binding of LASP1 to these proteins, is conceivable. In all, LASP1 is more than just a simple F-actin binding protein.

## Data Availability

Not applicable.
